# Comprehensive molecular profiling of the African swine fever virus in Korean wild boars between 2019 and 2024

**DOI:** 10.1186/s13567-026-01723-z

**Published:** 2026-05-13

**Authors:** Garam Kim, Sungiin Ji, Sua Choi, Seungmin Lim, HanTer Choi, Moon Jeong, Seondong Park, Weon-Hwa Jheong

**Affiliations:** Wildlife Disease Response Team, National Institute of Wildlife Disease Control and Prevention (NIWDC), 1 Songam-Gil, Gwangsan-Gu, Gwangju, Republic of Korea

**Keywords:** African swine fever virus, wild boar, molecular epidemiology, genetic diversity, transmission dynamics, South Korea, multi-marker analysis

## Abstract

African swine fever (ASF), caused by the African swine fever virus (ASFV), is a lethal transboundary disease. Since its first detection in South Korea in 2019, ASFV has continuously infected wild boars, imposing major economic and ecological burdens. We investigated ASFV genetic diversity and transmission dynamics by analyzing 4,209 ASFV-positive wild boar samples collected between 2019 and 2024 using a multi-marker approach, including *B646L* (p72), the intergenic region (IGR) between I73R/I329L, MGF 360-1La, and MGF 505-9R/10R. All isolates were classified as Genotype II by p72 analysis. IGR II was predominant, while one case of IGR I and three of IGR III were detected in 2019–2020. IGR III reappeared in 2023 and increased in 2024. The Korean-specific MGF 360-1La mutation (L106P) emerged in 2020, peaked in 2021, and persisted thereafter at a low frequency. A shift in the MGF 505-9R/10R profile was observed: MGF-1 dominated until 2020, whereas MGF-5 emerged in 2021 and became predominant in 2024. Accordingly, ASFV strains were classified into six clusters with distinct spatial distributions and transmission trajectories. Cluster 1 represented the initial incursion and early spread, and two minor subclusters (1.1 and 1.2) were detected early on; Cluster 2 remained localized, Cluster 3 expanded southeastward and is emerging as dominant, and Cluster 4 was confined to eastern Gyeongsangbuk-do. These findings suggest that although ASFV in Korea originated from a single Genotype II introduction, the detection of multiple lineages reflects viral diversification or additional incursions. Continuous molecular surveillance using genetic markers, complemented by whole-genome analyses, is essential for detecting early variants and developing effective ASF control strategies.

**Supplementary Information:**

The online version contains supplementary material available at 10.1186/s13567-026-01723-z.

## Introduction

African swine fever (ASF), caused by the African swine fever virus (ASFV), is a highly contagious and lethal viral disease that affects domestic pigs and wild boars, with a mortality rate approaching 100% [1, 2]. This disease poses a critical threat to the global swine industry. In South Korea, the first ASF outbreak was reported on September 16, 2019, at a pig farm in Paju, Gyeonggi-do [3]. On October 3, 2019, the first ASFV-infected wild boar was detected in the northwestern Demilitarized Zone [4]. Subsequently, ASF infection in wild boars has spread continuously in an eastward and southward direction [5]. In pig farms, 14 outbreaks occurred in 2019, followed by 2 in 2020, 5 in 2021, 7 in 2022, and 10 in 2023 [6]. These outbreaks have imposed a substantial socioeconomic burden on the Korean swine industry.

ASFV is an enveloped double-stranded DNA virus belonging to the genus *Asfivirus* within the family Asfarviridae [1, 7]*.* Similar to other double-stranded DNA viruses, the ASFV genome is highly conserved, and nucleotide substitutions, which are the main driver of viral evolution, occur at a relatively slow rate [8]. In addition, naturally occurring deletions in specific genomic regions have been reported in several field isolates and may also contribute to ASFV evolution and phenotypic changes [9]. Therefore, analyzing ASFV genetic variation, including both nucleotide substitutions and deletions, using specific molecular markers is essential for understanding viral evolution and transmission pathways [9, 10]. Sequencing of the carboxyl terminus of *B646L* (p72) represents the common standard marker for classifying ASFV into 24 distinct genotypes [11, 12], forming the basis for the global phylogenetic classification of the virus. Genotype I was the first to exhibit intercontinental dispersion and was introduced from West Africa to Portugal in 1957 and 1960, subsequently spreading to other European countries, South America, and the Caribbean [2, 13]. In 2007, genotype II ASFV was introduced from southern Africa into Georgia, initiating its rapid spread across Eastern Europe [14]. The virus subsequently disseminated to Armenia (August 2007) and the Russian Federation (November 2007), and was first reported in China in 2018 [15]. From China, genotype II ASFV rapidly expanded into neighboring Asian countries, eventually reaching South Korea in 2019 [16–19].

Although *B646L* aids in ASFV genotype classification, the high genetic homogeneity of Genotype II viruses limits its application in tracing virus introduction sources, transmission routes, or evolutionary dynamics [20]. To overcome these limitations**,** additional molecular markers have been identified and applied. For example, the tandem repeat sequences (TRS) in the intergenic regions (IGR) between I73R/I329L and MGF 505-9R/10R have been used to differentiate Genotype II subgroups circulating in Eastern Europe and Asia [10, 21–25]. In particular, the intergenic region between the I73R and I329L genes has been widely used as a standard subtyping marker for Genotype II ASFV, allowing the classification of viruses into four variants, designated IGR I, II, III, and IV [26]. In addition, single-nucleotide polymorphisms in O174L, K145R, and MGF 505-5R have been adopted to distinguish regional clusters in countries such as Poland, Romania, and Germany [24, 25, 27]. In Vietnam, an 11-bp insertion within the A179L/A137R IGR was reported, providing a basis for the further subdivision of Genotype II viruses [28].

While each marker provides valuable insights, their resolution is limited when used individually. Therefore, recent studies have adopted multi-marker approaches, integrating several genetic markers to achieve high-resolution molecular epidemiological analyses. In Europe, the multi-marker analysis of 382 ASFV isolates collected between 2007 and 2022 using the central variable region (CVR), IGRs (I73R/I329L; MGF505-9R/10R), O174L, and K145R enabled subdivision into 24 genetic clusters, which facilitated the identification of new introduction events and detailed transmission dynamics [24]. Similarly, in Italy (2022–2023), the combined application of CVR, IGR (I73R/I329L), O174L, and K145R markers revealed dominant groups and newly emerging clusters, allowing for the evaluation of epidemiological links between wild boars and domestic pig cases [29]. These studies demonstrate that multi-marker analysis is a powerful tool for resolving ASFV transmission pathways and regional connections at high resolution. However, a systematic multi-marker analysis of ASFV in Korean wild boars remains limited.

In South Korea, molecular marker-based analyses of ASFV detected in wild boars have been conducted continuously since 2019. These investigations revealed diverse mutation combinations within Genotype II, illustrating the ongoing formation and dissemination of new viral lineages over time. In this study, we applied a multi-marker approach using four genetic markers (p72, IGR, MGF 360-1La, and MGF 505-9R/10R) to classify ASFV strains detected in wild boars between 2019 and 2024 into six genetic clusters, comprising four major clusters and two minor subclusters. Through this analysis, we aimed to elucidate the spatial distribution, transmission routes, and emergence of novel lineages of ASFV in South Korea to provide fundamental insights into effective disease control and management strategies.

## Materials and methods

### Spatial analysis of ASF occurrences and genetic groupings from 2019 to 2024

Between 2019 and 2024, a total of 4,209 ASFV-positive wild boar cases were included in the spatial analysis, and their outbreak locations were mapped using ArcGIS Pro version 3.2 (Esri, Redlands, CA, USA) and QGIS version 3.4. Spatial distribution and movement patterns were visualized based on the geographical coordinates of each confirmed case. Genetic grouping was performed only for samples with complete molecular marker information, and these marker-defined clusters were subsequently overlaid onto the spatial dataset for visualization.

### Detection of the ASFV in wild boar samples

DNA was extracted from wild boar blood samples using the Maxwell RSC Viral Total Nucleic Purification Kit (Promega, Madison, WI, USA), following the manufacturer's protocol. Real-time PCR was performed using the commercially available VetMAX™ African Swine Fever Virus Detection Kit (Thermo Fisher Scientific, USA) following the manufacturer’s instructions, with 5 µL of extracted DNA. ASFV was detected in the FAM channel, while the internal positive control (IPC) was detected in the VIC channel. The IPC was used to monitor the quality of the extracted nucleic acid and to detect potential PCR inhibition. All reactions were performed using an Applied Biosystems QuantStudio 5 Real-Time PCR System (Thermo Fisher Scientific, Paisley, UK). Conventional PCR was conducted using the B646L (F: 5′-GGCACAAGTTCGGACATGT-3′, R: 5′- GTACTGTAACGCAGCACAG-3′) [11] and PPA (5′- AGTTATGGGAAACCCGACCC-3′, R: 5′- CCCTGAATCGGAGCATCCT-3′) [30] primers targeting regions within the ASFV *B646L* (P72) gene. Conventional PCR amplification was performed using an Applied Biosystems ProFlex System, and the resulting PCR products were purified and sequenced using Sanger sequencing (Daejeon, Korea).

### PCR amplification and sequencing of targeted genes

PCR amplification of ASFV was conducted using the primers detailed in Table 1. The target genes were amplified using a BioFACT 2X Multi-Star PCR series DNA polymerase kit (BioFACT, Daejeon, Korea). The annealing temperatures for PCR were set to 60 °C for B646L and PPA, 58 °C for MGF 360-1La, 56 °C for I73R/I329L, and 56 °C for MGF 505-9R/10R. The amplified PCR products were electrophoresed on a 1.5% agarose gel (BioFACT, Daejeon, Korea) and compared with a 1-kb DNA plus ladder (BioFACT, Daejeon, Korea) to verify successful amplification. A separate aliquot of each PCR reaction was submitted to BioFACT for enzymatic purification and Sanger sequencing. Raw Sanger chromatogram files were imported into Geneious Prime version 2025.1.2 (Biomatters Ltd., Auckland, New Zealand). Base calls and quality scores were inspected, and low-quality bases at the 5′ and 3′ ends were trimmed using the built-in quality trimming function (error probability limit of 0.01, approximately corresponding to Phred Q20). For each amplicon, the reverse read was reverse-complemented and aligned with the corresponding forward read in Geneious Prime, and a single consensus sequence was generated from the overlapping region. When discrepancies between the two reads were observed, the chromatograms were re-examined, and the base with the higher peak quality and Phred score was selected for the final sequence.

**Table 1:** **Characteristics of the primers used in this study.**

Target		Primer sequence (5′–3′)	Amplicon	Reference
B646L	F	GGCACAAGTTCGGACATGT	478 bp	Bastos et al. [11]
	R	GTACTGTAACGCAGCACAG		
I73R/I329L	F	CCATTTATCCCCCGCTTTGG	336 bp	Rodriguez et al. [26]
	R	TCGTCATCCTGAGACAGCAG		
MGF 360-1L	F	CCGATTAATGTCAGCCCCCA	540 bp	Kim et al. [5]
	R	TGCAGACATCAGCTTTGGGT		
MGF 505-9R/10R	F	ATTTAAGTAAACCATGTATATATCA	600 bp	Gallardo et al. [24]
	R	CTTAAGGCCTCGTTGATGGAAA		

### Phylogenetic analysis of ASFV

To determine the phylogenetic relationship of ASFV, multiple sequence alignments were performed using a fast Fourier transform algorithm in Geneious Prime version 2025.1.2 (accessed on July 29, 2024). Subsequently, a maximum likelihood (ML) phylogenetic tree was constructed based on the complete genome of ASFV using the ClustalW tool in Molecular Evolutionary Genetics Analysis (MEGA) 11 to evaluate the reliability of the tree topology [31]. To assess the robustness of the phylogenetic tree, ML bootstrap analysis was conducted using 1000 replicates.

### Complete ASFV genome sequencing and analysis

Library preparation was performed using an enzymatic library preparation kit (Celemics, Seoul, Republic of Korea) after shearing the genomic DNA (gDNA). The gDNA library was then hybridized with capture probes using a target enrichment kit (Celemics), which contains chemically synthesized probes designed to hybridize to the target regions. Post-PCR amplification was conducted to enrich the captured fragments. The target-enriched library was sequenced using the Illumina NextSeq 550 platform (Illumina, San Diego, CA, USA) with a 2 × 150 bp paired-end read configuration. Adapter sequences and low-quality bases were removed using the Fastx Toolkit 0.0.14, and additional trimming was performed using AdapterRemoval version 2.2.2. Cleaned reads were mapped to the reference ASFV genome (accession number: FR682468) using Burrows–Wheeler Aligner (BWA) version 0.7.10. Single-nucleotide polymorphisms (SNPs), insertions and deletions (indels), and structural variations were identified using the Genome Analysis Toolkit (GATK) version 4.0.4.0. Read alignment quality was assessed using SAMtools (version 1.1) and the Python package NumPy (version 1.11.0).

### Genetic clustering and directional movement analysis

In this study, genetic and spatial data from 4,209 ASFV-positive wild boar cases detected in South Korea between 2019 and 2024 were analyzed. Each sample was classified into one of six genetic groups (Clusters 1, 1.1, 1.2, 2, 3, and 4) based on the combination of four molecular markers: the IGR, p72 genotype, MGF 360-1La variant, and the TRS between MGF 505-9R and 10R. All samples were subjected to molecular marker analysis; however, some yielded incomplete genetic information or produced unclassifiable marker combinations. Because cluster assignment required complete information for all four loci, approximately 70% of the samples collected during 2019–2024 could be assigned to a genetic cluster. Standard deviational ellipses were generated to visualize the spatial dispersion of each genetic group. These ellipses were calculated based on the covariance matrix of the x and y coordinates, using the mean center of each group as a reference [32]. The lengths and orientations of the major and minor axes represent the direction and spatial spread of the ASFV cases within each group. This analysis was performed using spatial statistical tools in ArcGIS Pro (Esri, Redlands, CA, USA).

To assess temporal movement trends, sample data were grouped by year, and a spatial median center was calculated for each cluster-year subset. The median center minimizes the total Euclidean distance to all features in the group and is less sensitive to outliers than the mean center. It is calculated using an iterative algorithm that minimizes the sum of distances to all points [33]. Next, the yearly median centers were connected chronologically to visualize the movement trajectories. Subsequently, movement direction was analyzed using the Line Bearing function within the Calculate Geometry Attributes tool in ArcGIS Pro. This function calculates the azimuth (bearing) between the start and end points of each line segment, measured clockwise from true north, with values ranging from 0° to 360° (e.g., 0° = North, 90° = East, 180° = South [34], 270° = West) [35].

The bearing (β) was calculated using the following equations [34]:

$$^\circ \,\beta \, = {\text{ arctangent }}\left( {{\text{Y }}/{\text{ X}}} \right)$$

where:

$$^\circ {\text{X }} = {\text{ cos}}\left( {\varphi {\mathrm{B}}} \right) \, \times {\text{ sin}}\left( {\Delta {\mathrm{L}}} \right)$$

$$^\circ {\text{Y }} = {\text{ cos}}\left( {\varphi {\mathrm{A}}} \right) \, \times {\text{ sin}}\left( {\varphi {\mathrm{B}}} \right) \, - {\text{ sin}}\left( {\varphi {\mathrm{A}}} \right) \, \times {\text{ cos}}\left( {\varphi {\mathrm{B}}} \right) \, \times {\text{ cos}}\left( {\Delta {\mathrm{L}}} \right)$$

φA and φB are the latitudes of the start and end points, respectively. ΔL is the difference in longitudes.

Each calculated bearing was categorized into any of the following directional sectors: 

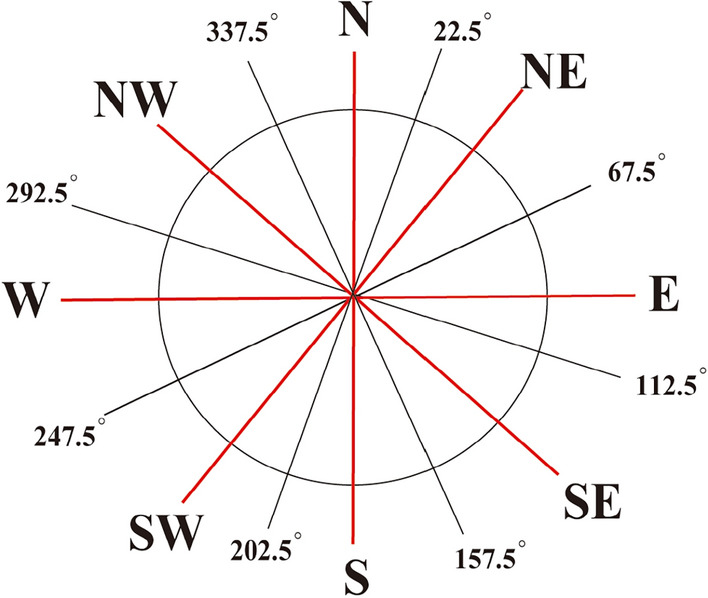



337.5° ≤ β < 22.5° → North (N); 22.5° ≤ β < 67.5° → Northeast (NE); 67.5° ≤ β < 112.5° → East (E); 112.5° ≤ β < 157.5° → Southeast (SE); 157.5° ≤ β < 202.5° → South (S); 202.5° ≤ β < 247.5° → Southwest (SW); 247.5° ≤ β < 292.5° → West (W); 292.5° ≤ β < 337.5° → Northwest (NW)

This approach enables a standardized and consistent interpretation of directional movement patterns for each ASFV genetic group across years.

## Results

### ASF occurrence status in wild boars between 2019 and 2024

**Figure 1:**
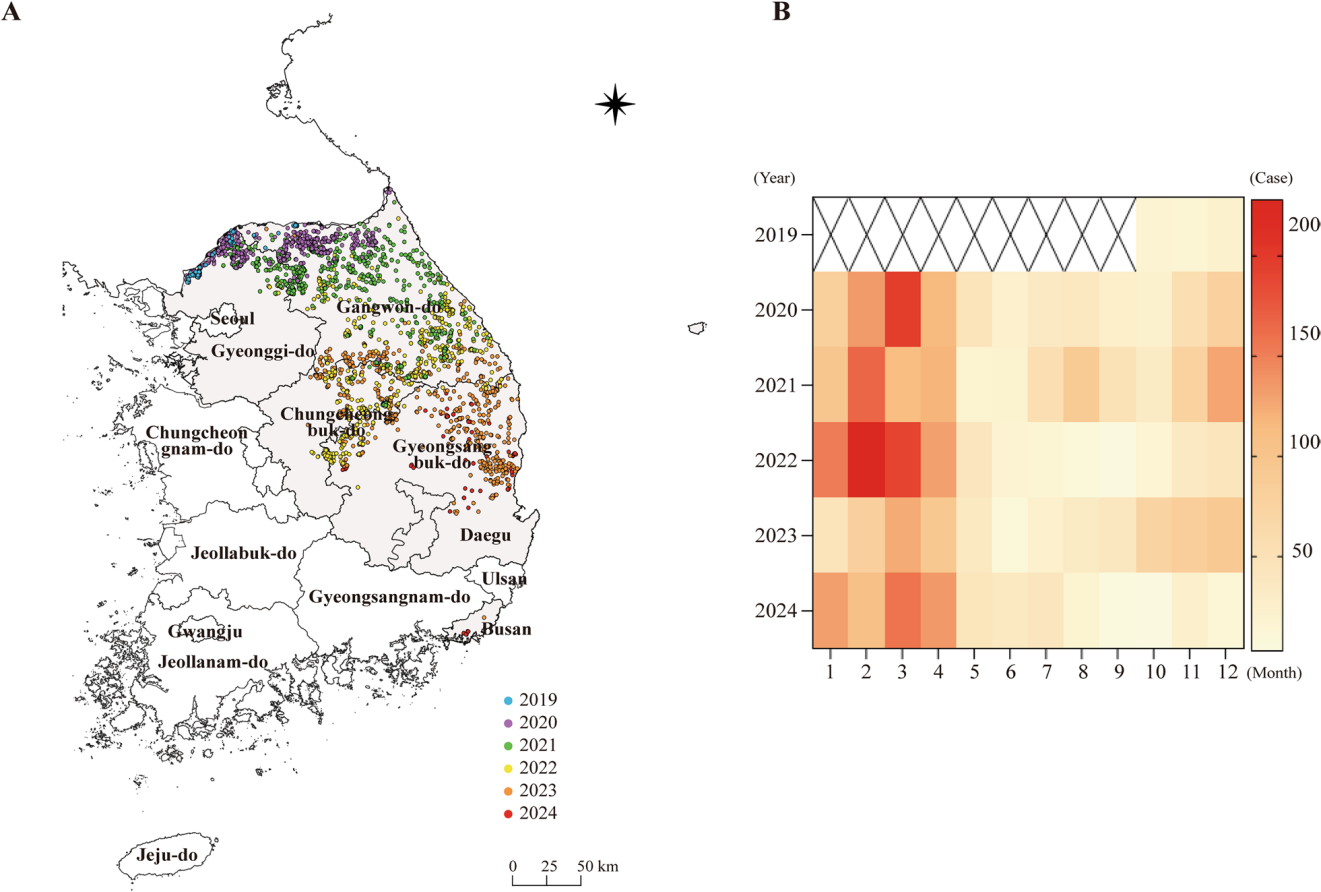
**Distribution of African swine fever (ASF) cases in wild boars in South Korea (2019–2024).** (**A**) The geographic distribution of the African swine fever virus (ASFV) in wild boars by year. Each colored dot represents the time and location of detection: light blue for 2019, purple for 2020, green for 2021, yellow for 2022, orange for 2023, and red for 2024. (**B**) A heatmap showing the number of ASFV-positive wild boar cases by year and month. Darker colors indicate a higher number of positive cases.

In October 2019, ASF was first detected in wild boars in Yeoncheon, South Korea [3] (Figure 1). Subsequently, ASF outbreaks in wild boars continued until 2024. Cases were initially concentrated in the northern regions, such as Gangwon-do and Gyeonggi-do, and gradually expanded to southern areas, including Gyeongsangbuk-do and Busan (Figure 1A). ASF cases were primarily reported between January and March, peaking in March 2020 and March 2022 (Figure 1B). In all years, the number of ASFV-positive cases showed a marked decline between June and October (Figure 1B).

### *B646L* (P72): genotyping marker

**Figure 2:**
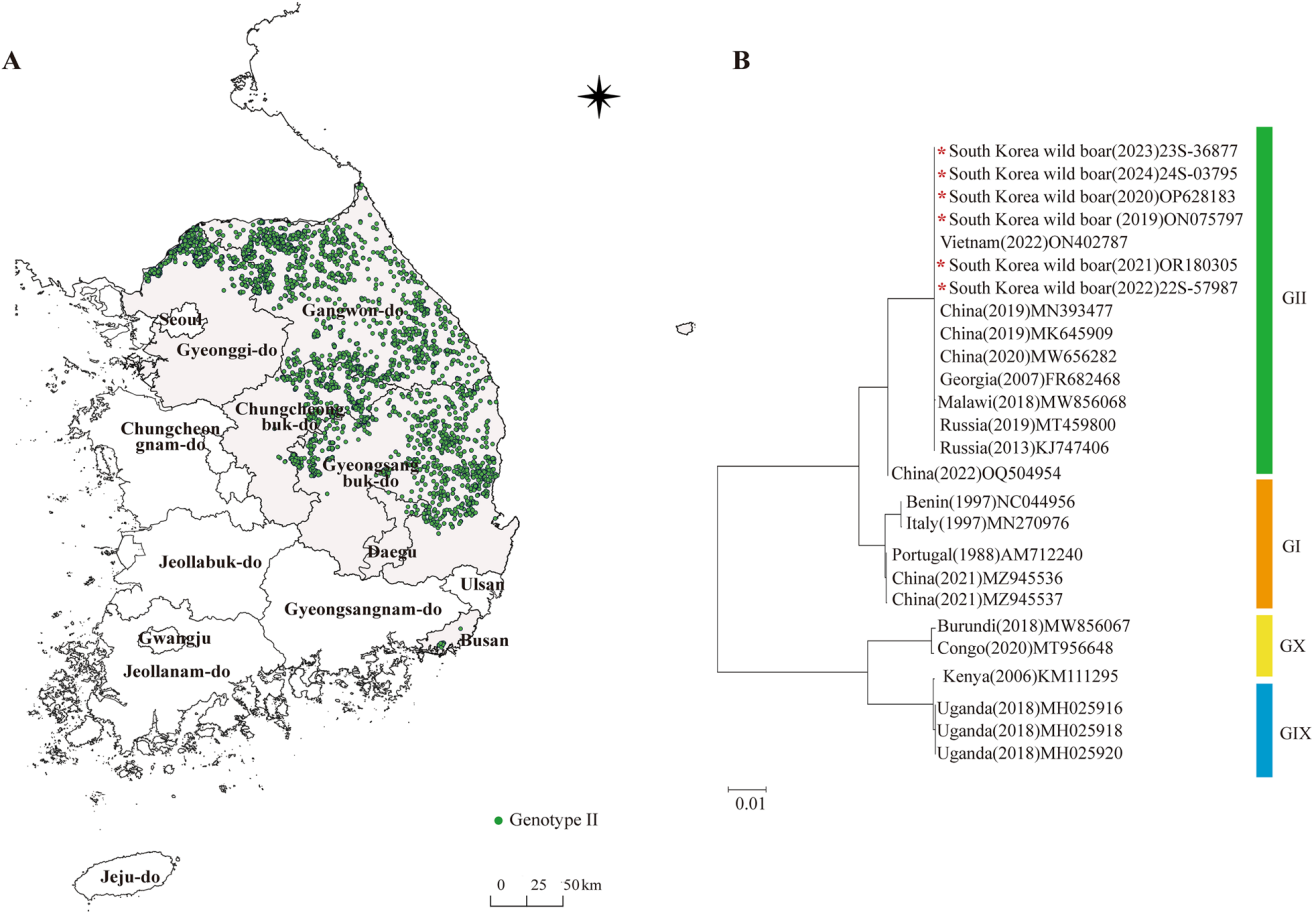
**Geographic distribution and phylogenetic classification of ASFV genotype detected in wild boars.** (**A**) The spatial distribution of ASFV genotype detected in wild boars across South Korea between 2019 and 2024. Green dots indicate confirmed Genotype II cases. (**B**) The phylogenetic tree constructed using complete genome sequences of the ASFV strains. Colored bars represent genotype classifications: green for Genotype II (GII), orange for Genotype I (GI), yellow for Genotype X (GX), and blue for Genotype IX (GIX). Red asterisks indicate ASFV sequences obtained from Korean wild boars (this study).

The first protocol for ASFV genotyping, based on PCR amplification and Sanger sequencing of the variable C-terminus of the *B646L* (p72), was established in 2002 [11]. Partial sequencing of *B646L* has classified ASFV strains into 26 globally recognized genotypes [36, 37]. In South Korea, 4209 ASFV-positive wild boar samples were collected between 2019 and 2024, and continuous molecular surveillance was conducted using *B646L* analysis. The genotyping coverage rate exceeded 95% across all regions, with the highest rate observed in Gyeonggi-do (98.2%) and the lowest in Busan (92.0%) (Table 2). All ASF outbreak regions, including Gangwon-do, Gyeonggi-do, Chungcheongbuk-do, Gyeongsangbuk-do, Daegu, and Busan, were confirmed to be associated with Genotype II (Figure 2A). Furthermore, phylogenetic analysis using whole-genome sequences of representative ASFV isolates from Korean wild boars collected between 2019 and 2024 confirmed that all Korean strains clustered within the Genotype II together with global Genotype II reference strains (Figure 2B).

**Table 2:** **Regional distribution and p72 analysis rates derived from ASF-positive wild boar samples (2019–2024).**

Region	Positive samples	P72 analyzed	Analysis rate (%)
Gangwon-do	1925	1847	95.9
Gyeonggi-do	676	664	98.2
Chungcheongbuk-do	527	511	96.9
Gyeongsangbuk-do	1038	1,007	97.0
Busan	25	23	92.0
Daegu	18	17	94.4
Total/Average	**4209**	**4069**	**95.8**

### I73R/I329L: subgenotyping marker

**Figure 3:**
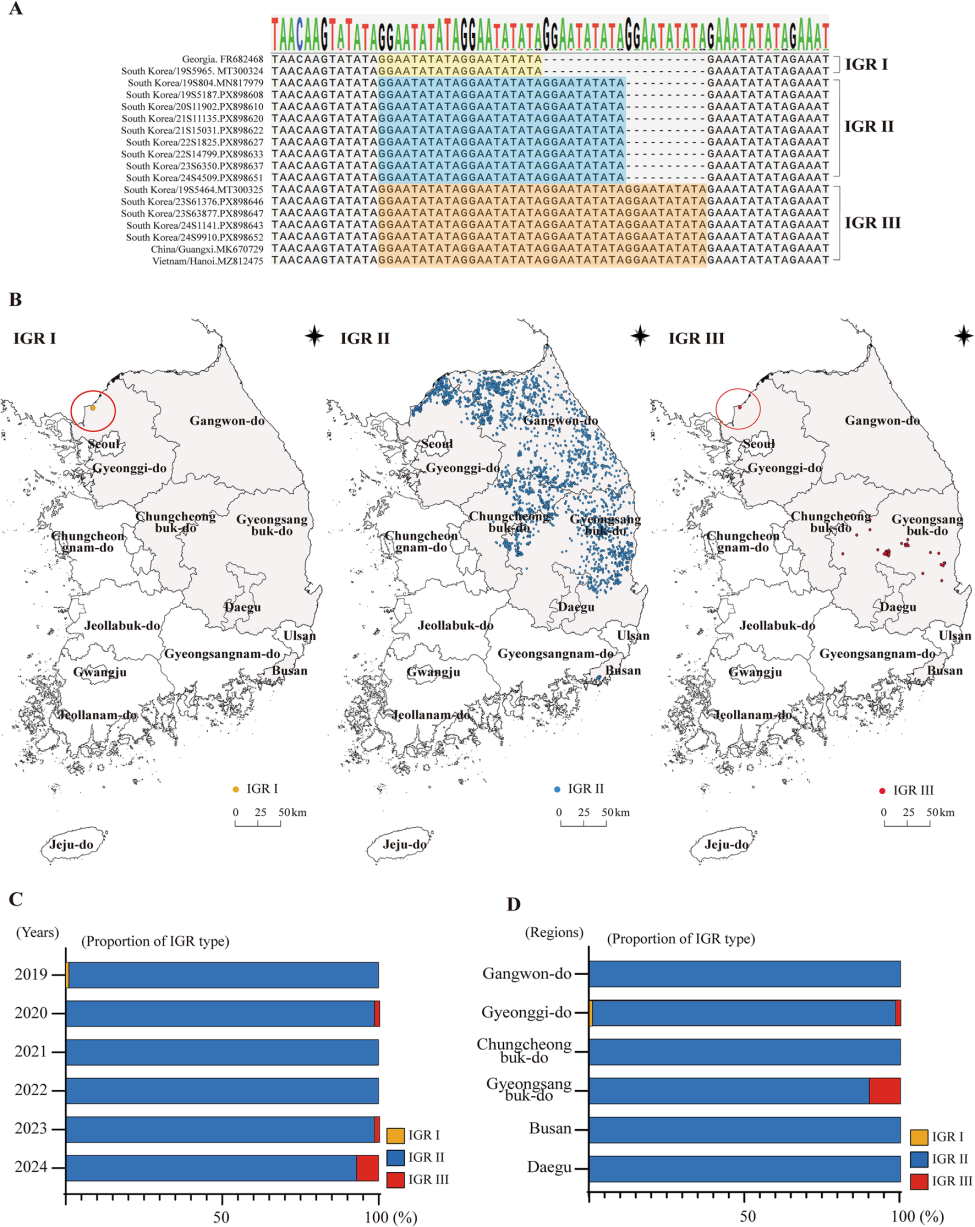
**Spatiotemporal distribution and annual proportions of ASFV intergenic region (IGR) variants detected in wild boars.** (**A**) The nucleotide sequence alignment of the intergenic region (IGR) between *I73R* and *I329L* detected in ASFV-positive wild boars in South Korea from 2019 to 2024. The three IGR variants IGR I, IGR II, and IGR III are shown, with their characteristic tandem repeat sequences (TRS) highlighted in yellow (IGR I), blue (IGR II), and red (IGR III), respectively. (**B**) The geographic distribution of the IGR types among ASFV-positive wild boars in South Korea (2019–2024). Each colored dot represents a confirmed IGR subtype: yellow for IGR I, blue for IGR II, and red for IGR III. (**C**) Annual proportions of the IGR types detected from 2019 to 2024. Each bar represents the relative frequency of IGR subtypes in a given year: yellow for IGR I, blue for IGR II, and red for IGR III. (d) Regional proportion of the IGR types. Each bar shows the relative frequency of IGR subtypes within different provinces. Yellow represents IGR I, blue represents IGR II, and red represents IGR III.

Sequence alignment of the intergenic region (IGR) between *I73R* and *I329L* revealed three distinct IGR types circulating in Korean wild boars: IGR I, IGR II, and IGR III (Figure 3A). These variants differed in the number of tandem repeat sequences (TRS), with IGR I containing one repeat unit, IGR II containing two units, and IGR III containing three units. Comparison with international reference strains demonstrated that the Korean IGR variants shared the same TRS based structural patterns as those previously reported in Georgia 2007/1 and Vietnamese isolates.

Having established the genetic characteristics of the three IGR types, we examined their temporal and spatial distributions to track the introduction and subsequent spread of ASFV Genotype II in Korean wild boars. In December 2019, one IGR I case and one IGR III case were detected in Paju, a northern border region (Figure 3B, left), followed by two additional IGR III cases in March 2020 at the same location (Figure 3B, right). From 2019 to 2024, most ASFV-positive samples were classified as IGR II (Figure 3B, center), and IGR I was not detected after its initial appearance. In contrast, IGR III re-emerged in 2023 and increased in frequency throughout 2024 (Figure 3C). Notably, whereas IGR I and early IGR III detections in 2019–2020 were confined to Gyeonggi-do, the IGR III variants that reappeared in 2023 and expanded in 2024 were found exclusively in Gyeongsangbuk-do (Figure 3D).

### MGF 360-1L: a unique subgenotyping marker in Korean wild boars

**Figure 4:**
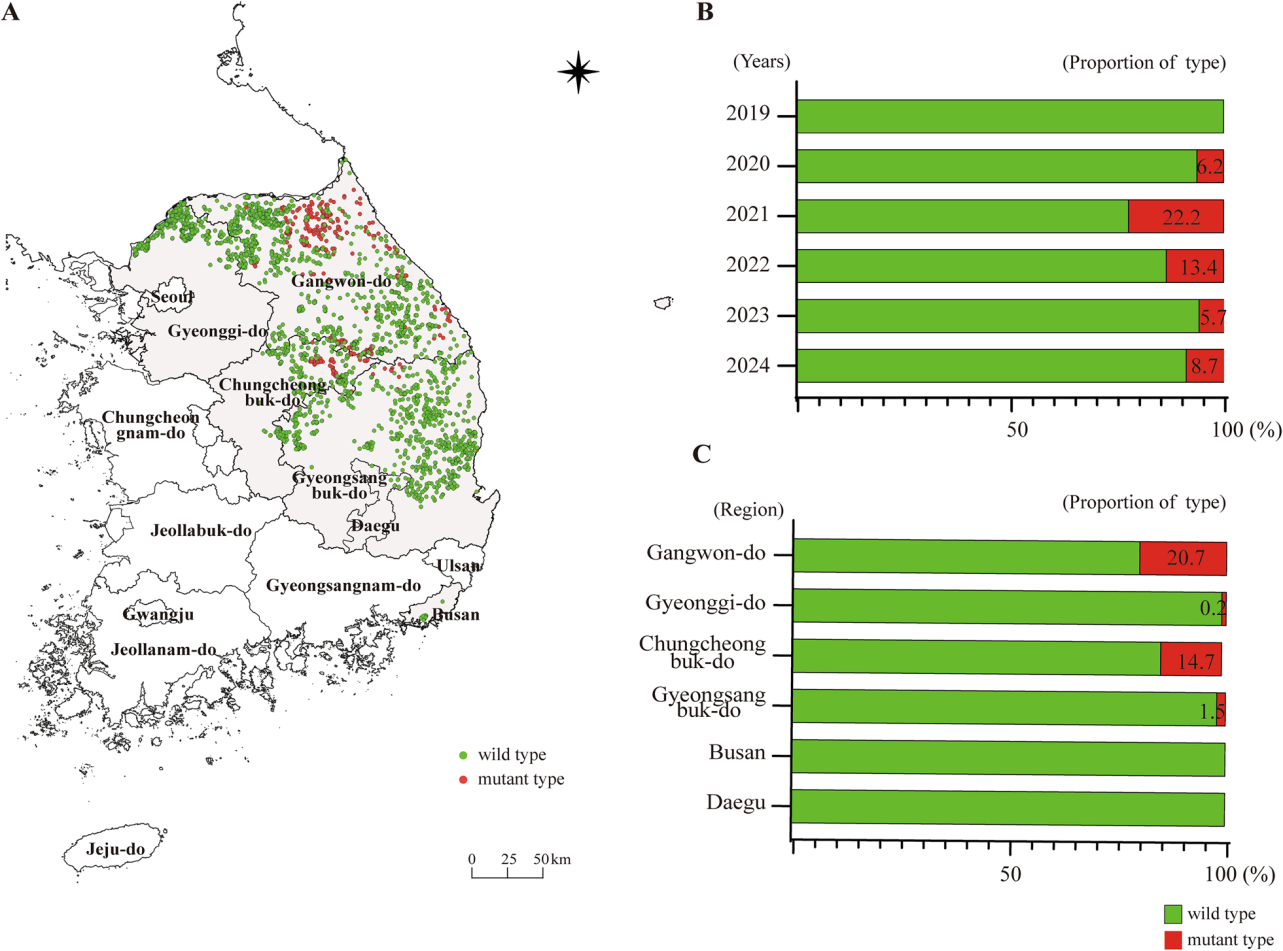
**Spatiotemporal distribution and annual proportions of wild-type (WT) and mutant-type (MT) MGF 360-1La variants. **(**A**) The spatial distribution of WT (green) and MT (red) MGF 360-1La. (**B**) Annual proportion of WT and MT MGF 360-1La in the ASFV between 2019 and 2024. (**C**) Regional proportions of WT and MT MGF 360-1La variants across affected provinces.

In 2020, a variation at amino acid position 106 of MGF 360-1La was identified in viruses isolated from ASF-infected wild boars in Korea. Strains with leucine (L) at this position were classified as wild-type (WT), whereas those with proline (P) were classified as mutant-type (MT) [5]. In this study, we used this L106P substitution in MGF 360-1La as a genetic marker, as it has been reported exclusively in Korean wild boars. Using this marker, we investigated the spatial distribution of ASFV from 2019 to 2024. Yearly trends showed that the mutant type was first detected in 2020, accounting for 6.2% of all cases. Its proportion increased markedly to 22.2% in 2021 and 13.4% in 2022. The frequency then declined to 5.7% in 2023 and 8.7% in 2024, while the wild type remained the predominant variant throughout the study period (Figure 4B). Regional trends indicated that the mutant type was most frequently detected in Gangwon-do (20.7%) and Chungcheongbuk-do (14.7%), whereas its proportion was very low in Gyeonggi-do (0.2%) and Gyeongsangbuk-do (1.5%). No mutant-type cases were identified in Busan or Daegu. These findings suggest that the mutant variant exhibited a geographically restricted distribution, occurring primarily in the central and northeastern regions of South Korea (Figure 4C).

### MGF 505-9R/10R: subgenotyping marker

**Figure 5:**
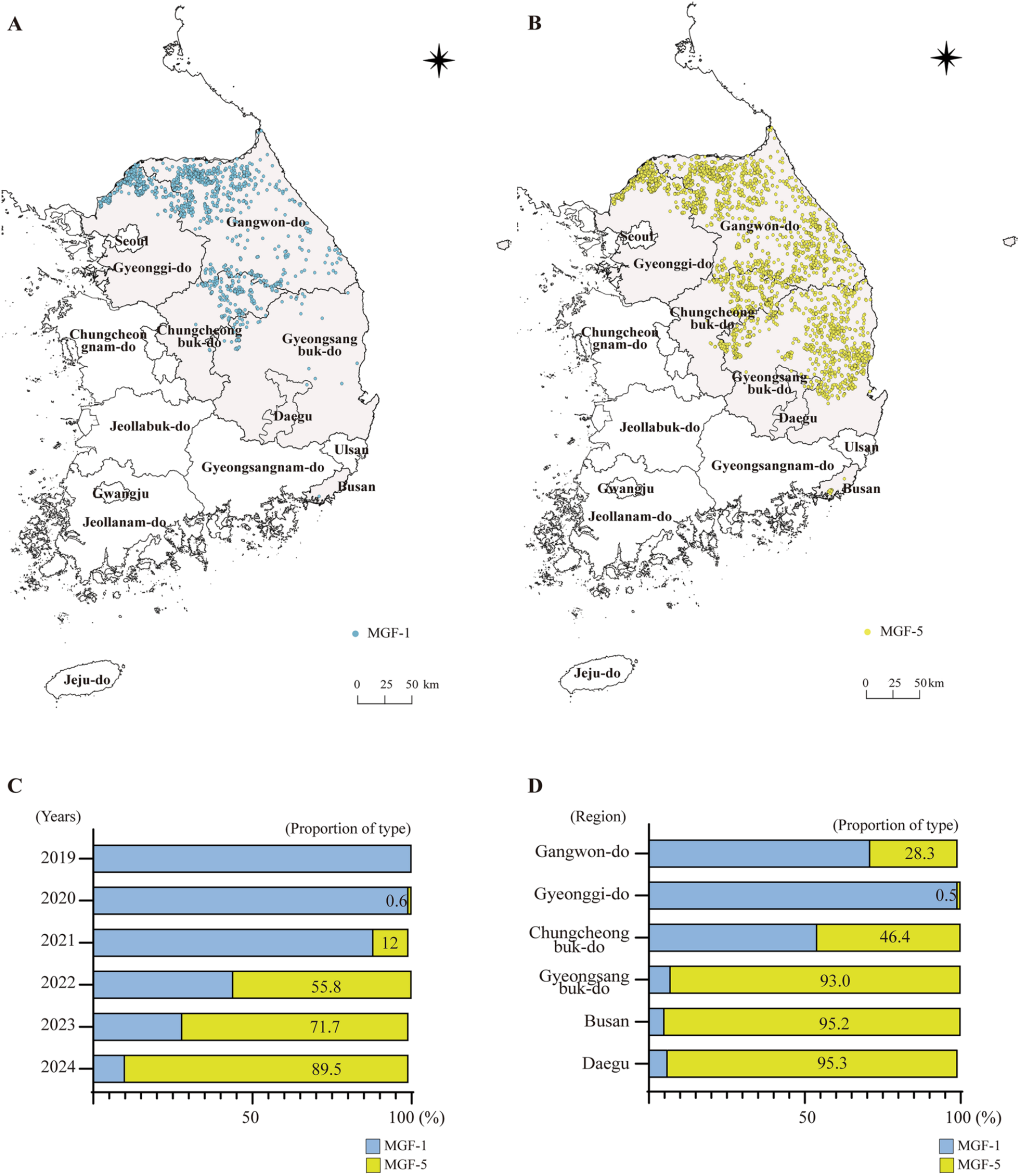
**Spatiotemporal distribution of ASFV subgenotypes MGF-1 and MGF-5 in wild boars in South Korea.** (**A** and **B**) The geographic distribution of ASFV subgenotypes MGF-1 (A, blue) and MGF-5 (B, yellow) across South Korea between 2019 and 2024. (**C**) Annual proportion of MGF-1 and MGF-5 subgenotypes among total ASFV-positive wild boar cases. (**D**) Regional distribution of MGF-1 and MGF-5 subgenotypes in major provinces.

Genotype II ASFV isolates have been classified into eight subgenotypes (MGF-1 to MGF-8) based on variations in the tandem repeat sequences within the MGF 505-9R/10R region [24]. Using this established classification framework, we analyzed ASFV detected in Korean wild boars to characterize the spatial and temporal dynamics of circulating variants between 2019 and 2024. In the distribution maps, MGF-1 is shown in blue and MGF-5 in yellow. MGF-1 was primarily distributed in central-northern regions, including areas near the North Korean border, whereas MGF-5 exhibited a broader distribution extending into central and southern regions (Figures 5A and 5B). Yearly trends showed that only MGF-1 was detected in 2019 (100%), while MGF-5 was first identified in 2020, accounting for 0.6% of cases. The proportion of MGF-5 subsequently increased to 12.0% in 2021, 55.8% in 2022, and 71.7% in 2023. By 2024, MGF-5 had become the predominant type, representing 89.5% of all samples (Figure 5C). Regional analysis also revealed marked differences in the distribution of MGF-1 and MGF-5 (Figure 5D). In Gangwon-do, MGF-5 accounted for 28.3% of cases, whereas its proportion in Gyeonggi-do remained very low at 0.5%. In contrast, the central and southeastern regions showed notably higher proportions of MGF-5, reaching 46.4% in Chungcheongbuk-do, 93.0% in Gyeongsangbuk-do, 95.2% in Busan, and 95.3% in Daegu. These findings suggest that the spread of the MGF-5 variant became increasingly concentrated in the southeastern regions of South Korea.

### Geographic distribution of the ASFV genetic clusters identified through molecular marker combinations

**Figure 6:**
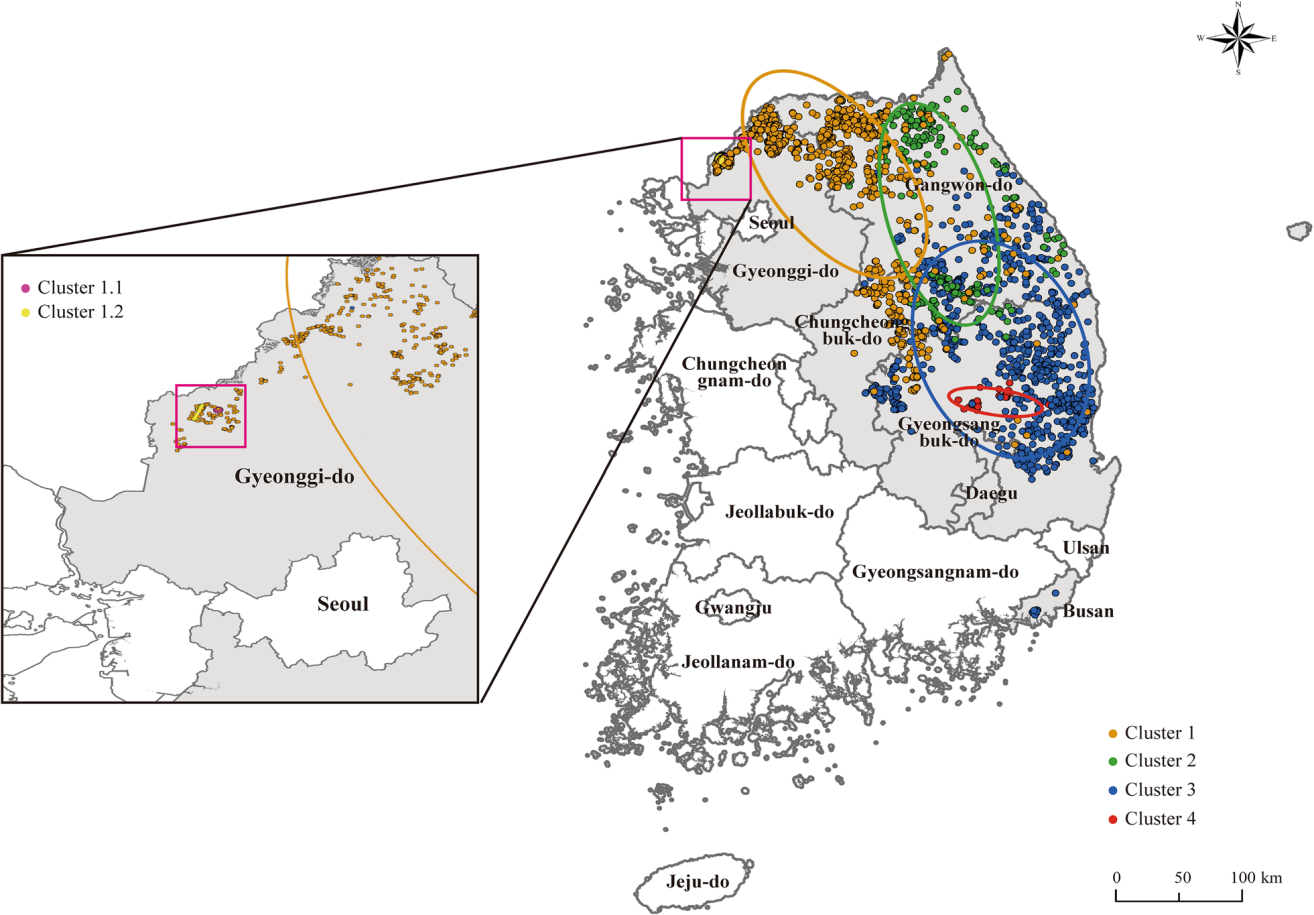
**Spatial distribution of ASFV clusters classified by genetic markers.** The map shows the spatial distribution of six genetic clusters (Clusters 1, 1.1, 1.2, 2, 3, and 4) of ASFV in wild boars, defined based on molecular markers B646L, IGR, MGF 360-1La, and MGF 505-9R/10R. Each dot represents a confirmed ASF case in wild boars and is color-coded by cluster: Cluster 1 (orange, *n* = 1340), Cluster 1.1 (yellow, *n* = 1), Cluster 1.2 (pink, *n* = 3), Cluster 2 (green, *n* = 330), Cluster 3 (blue, *n* = 1299), and Cluster 4 (red, *n* = 40).

To classify the ASFV isolates into distinct genetic clusters, we analyzed combinations of four molecular markers**:** B646L genotype**,** IGR variant, amino acid residue at position 106 in MGF 360-1La, and TRS of the MGF 505-9R/10R region. Each cluster was defined by a unique combination of these markers, as summarized in Table 3, and a schematic visualization of these combinations is additionally presented in Table 4 to provide a clear depiction of how the four markers collectively distinguish the six clusters identified in this study. Between 2019 and 2024, the ASFV-positive cases detected in wild boars were classified into six genetic clusters (Clusters 1, 1.1, 1.2, 2, 3, and 4) based on molecular markers, and their geographic distribution was visualized on a map (Figure 6). Cluster 1 (orange) was predominantly detected in northern Gyeonggi-do and Gangwon-do near the North Korean border and was the dominant cluster during the early phase of the ASFV outbreak in South Korea. Clusters 1.1 (pink) and 1.2 (yellow) were identified in northern Gyeonggi-do and classified as IGR I and III, respectively. Each cluster was detected in only one (Cluster 1.1) and three (Cluster 1.2) cases, indicating a low detection frequency (left inset). No further cases belonging to these clusters have been identified. Cluster 2 (green) was characterized by a unique amino acid substitution (L106P) in the MGF 360-1La and was primarily detected in central Gangwon-do and northern Chungcheongbuk-do, suggesting a variant lineage with a limited transmission route. Cluster 3 (blue) possessed an MGF-5-type tandem repeat structure in the MGF 505-9R/10R region and was distributed across eastern Gangwon-do, Chungcheongbuk-do, and Gyeongsangbuk-do. This pattern may indicate an emerging and actively spreading new lineage. Cluster 4 (red) shared the IGR III variant with Cluster 1.2; however, it exhibited a distinct combination of molecular markers and was geographically restricted to eastern Gyeongsangbuk-do, suggesting that it represents a genetically independent lineage.

**Table 3:** **Key molecular marker combinations used to define ASFV genetic clusters.**

Genetic cluster	**Genetic marker**			
	B646L	IGR	MGF 360-1La (aa106)	MGF 505-9R/10R
Cluster 1	II	II	L	MGF-1
Cluster 1.1	II	I	L	MGF-1
Cluster 1.2	II	III	L	MGF-1
Cluster 2	II	II	P	MGF-1
Cluster 3	II	II	L	MGF-5
Cluster 4	II	III	L	MGF-5

**Table 4:**
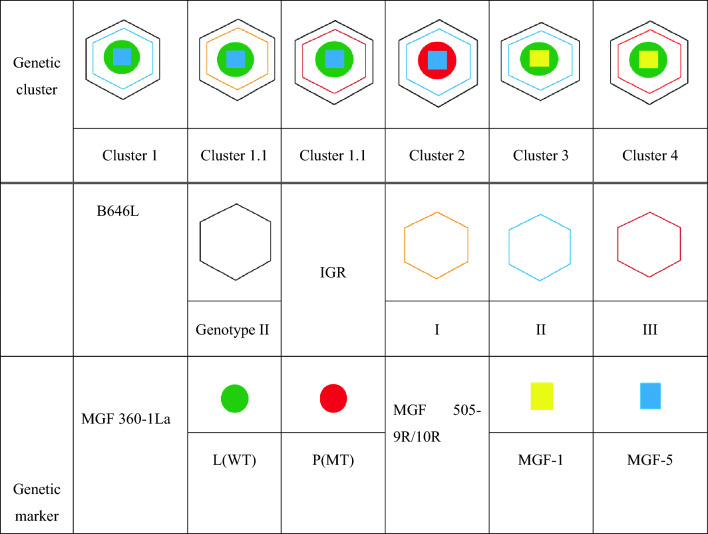
**Schematic representation of key molecular marker combinations used to define ASFV genetic clusters.**

### Estimated transmission routes of the ASFV genetic clusters in South Korean wild boars (2019–2024)

**Figure 7:**
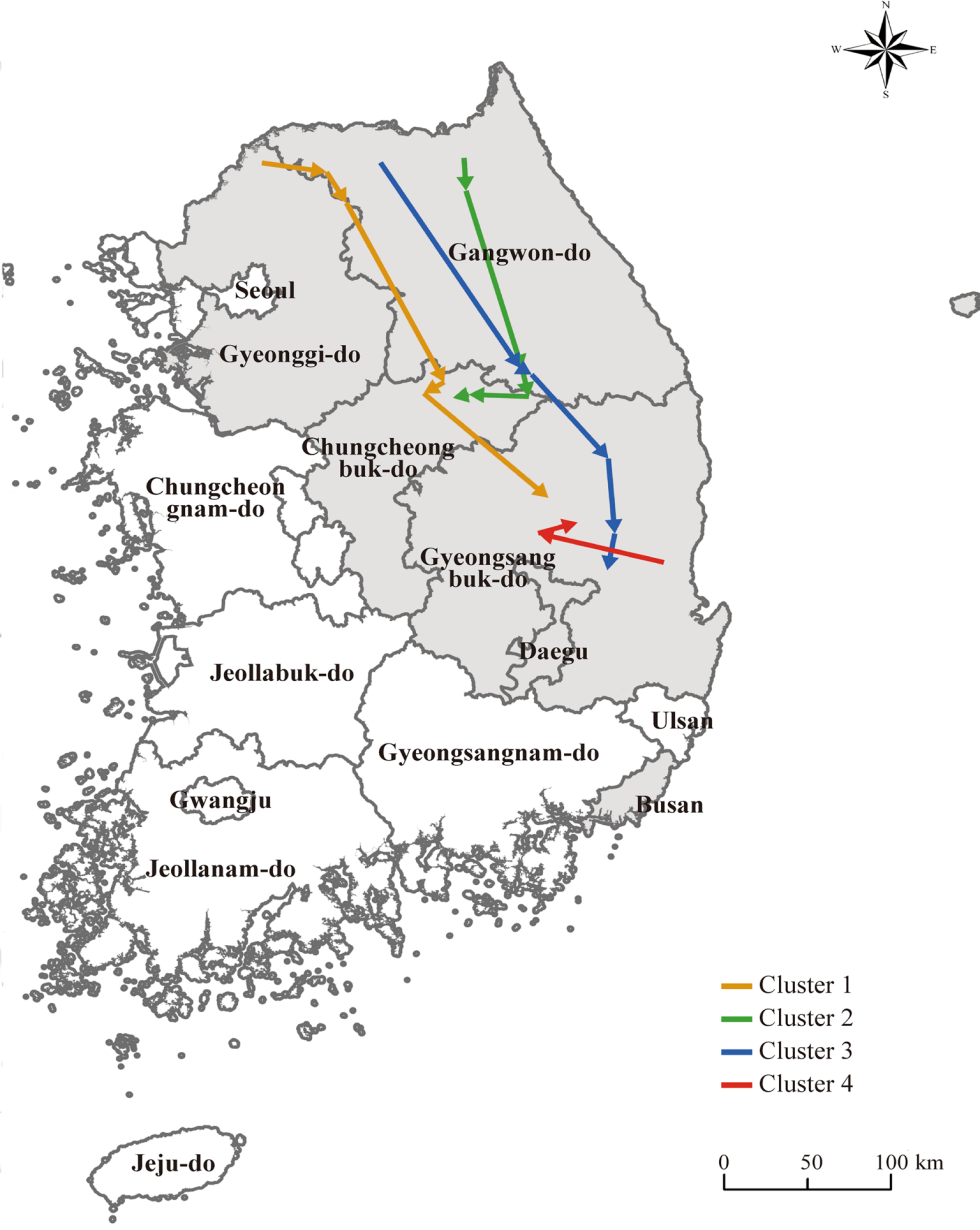
**Geographic spread patterns of ASFV molecular clusters identified in wild boars between 2019 and 2024.** This map illustrates the spatial distribution and inferred transmission directions of four major ASFV molecular clusters detected in wild boars between 2019 and 2024. Clusters 1, 2, 3, and 4 are shown in orange, green, blue, and red, respectively, on the map.

The ASF cases in wild boars between 2019 and 2024 were analyzed based on molecular clustering, revealing distinct transmission directions for each cluster (Figure 7). Cluster 1 (orange) exhibited a clear southward spread. It is presumed to have originated in the northern regions of Gyeonggi-do and Gangwon-do, near the North Korean border, and gradually expanded into inland areas such as Chungcheongbuk-do and northern Gyeongsangbuk-do. This cluster appears to have formed the primary transmission route during the early phase of ASFV spread in South Korea. Cluster 2 (green) was confined to central Gangwon-do and northern Chungcheongbuk-do, with no evidence of spread beyond these areas, indicating a localized transmission pattern. Cluster 3 (blue) originated from northern Gangwon-do and subsequently spread southeastward into central Gyeongsangbuk-do. This cluster showed limited geographic overlap with other clusters and is interpreted as a distinct lineage with an independent transmission route. Moreover, its detection frequency has increased since 2022, suggesting that it represents an emerging dominant cluster undergoing gradual expansion in the central-southern inland region. Cluster 4 (red) was initially detected in the eastern coastal area of Gyeongsangbuk-do and later spread westward into inland regions.

## Discussion

ASFV poses a critical threat to the global swine industry and wildlife health and continues to spread. ASF was first detected in wild boars in Yeoncheon, Korea, in October 2019, and outbreaks have persisted for approximately five years (Figure 1). These continuous outbreaks have caused substantial damage to the swine industry and wildlife health in the country. Although ASFV, the causative agent of ASF, is considered a relatively stable DNA virus [1], previous studies have reported that mutations, such as changes in TRS, amino acid substitutions, and small insertions or deletions, occur in specific genomic regions [38]. Furthermore, recent studies have increasingly reported the emergence of recombinant ASFV strains involving genotype I and genotype II viruses [39, 40]. These mutations represent significant molecular markers for subgenotyping and cluster differentiation, and they are practically useful for epidemiological investigations [24, 41]. Thus, beyond the simple diagnosis of ASF, genomic surveillance has been increasingly recognized as an essential tool for epidemiological tracing to elucidate its transmission routes and origins. In this study, a combination of the genetic markers *B646L* (p72)*,* IGR, MGF 360-1La, and MGF 505-9R/10R was selected to define the genetic clusters of the ASFV. This approach enabled us to reconstruct the temporal and spatial patterns of ASFV dissemination and infer possible transmission directions, thereby enhancing our understanding of ASFV transmission dynamics, supporting the interpretation of potential viral incursions or re-emergence, and informing effective control strategies.

ASF originated in Africa and has subsequently spread worldwide [42]. Therefore, tracing the connections among outbreaks in different countries and identifying the routes of introduction are of critical significance [43]. Viral genetic analysis is a key tool for such epidemiological investigations. Among various markers, *B646L* (p72) has become a significant marker for diagnosis and is a representative molecular marker for distinguishing ASFV genotypes. Bastos et al. classified ASFV genotypes based on p72 sequences, and it has now been reported that the virus comprises up to 26 genotypes [11, 37, 44]. In this study, all ASFV strains detected in Korean wild boars between 2019 and 2024 were identified as Genotype II (Figure 2). This finding indicates that since the initial introduction of ASFV into Korea in 2019, the same genotype has continued to circulate and spread, suggesting that the domestic epidemic has been maintained by a single genotype. Although the ASFV can be genotyped using p72, this approach has limited resolution for distinguishing lineages within the same genotype. Therefore, additional analyses of variable regions, such as EP402R (CD2v), the intergenic region (IGR), and the central variable region (CVR) within B602L, together with various region-specific unique markers, can capture genetic polymorphisms in finer detail and allow a more precise reconstruction of the phylogenetic relationships among circulating virus strains; these molecular markers have been used as important tools for epidemiological tracing and phylogenetic analyses [29, 41, 45, 46]. However, although EP402R and B602L-CVR have been monitored in parallel with the IGR in molecular surveillance of ASFV variants in Korean wild boars, no nucleotide variation has yet been detected in these regions among genotype II ASFV isolates from Korea.

The 10-bp TRS, located between the I73R and I329L genes, has been established as a standard genetic marker for subtyping Genotype II viruses. In the reference genome Georgia 2007/1 (FR682468.2), the IGR between I73R and I329L contains two copies of the 10-bp TRS, which is classified as IGR I. In 2014, Gallardo et al. identified an additional TRS insertion in ASFV isolates from Ukraine, Belarus, Lithuania, Poland, and Russia, which was subsequently designated as IGR II. Subsequently, IGR I and IGR II variants have been reported in Europe, Russia, and Asia, with IGR II becoming the predominant subtype in the European Union after 2014 [20, 24, 47–49]. IGR III, characterized by four TRS repeats, was first identified in Guangxi Province, China, in 2019, and additional cases of IGR III were subsequently reported in Vietnam in 2022 [50, 51]. The ASFV, first introduced into Korea in 2019, was identified as IGR II, which subsequently became the predominant IGR variant circulating in the country. In Paju, one case of IGR I and three of IGR III were detected in 2019 and 2020; however, IGR II remained the dominant type thereafter. In November 2023, IGR III re-emerged in Cheongsong and Pohang [52], and subsequent detections in nearby areas raised concerns about the potential for re-circulation of this subtype (Figure 3). These findings suggest the possibility of re-introduction, although viral mutations or the influence of multiple contributing factors cannot be excluded.

In ASFV strains from Korean wild boars, a substitution at amino acid position 106 of MGF 360-1La, where leucine is replaced by proline, has been identified and is considered a unique marker reported only in Korea [5]. This mutation was first detected in the northern region of Gangwon-do in 2021, was most frequently observed in 2022, and has persisted. Such a mutation could be a distinctive molecular marker for tracing lineage diversification and regional transmission pathways of ASF outbreaks in Korea.

Gallardo first proposed analyzing the TRS within MGF 505-9R/10R as a novel marker in addition to the traditional types [53]. This marker aids in classifying MGF groups into 1 to 8. In the reference genome Georgia 2007/1, as well as in the ASFV strains identified in Russia and most European regions, the viruses predominantly belonged to MGF-1, while other MGF groups were detected in certain regions [24]. Through continuous whole-genome surveillance, we first identified a novel variant of MGF 505-9R/10R in northern Gyeonggi-do in 2020. The viruses initially introduced and disseminated in 2019 belonged to MGF-1; however, in 2020, MGF-5 was detected for the first time in this region. Subsequently, the prevalence of MGF-5 has steadily increased, and the type became dominant in 2024, accounting for approximately 90% of the ASFV strains detected (Figure 6). In contrast, the ASFV strains from domestic pig farms in Korea during 2022–2023 belonged to MGF-1 [48]. In Europe, the MGF-5 (MGF-V) variant had previously been reported in a wild boar in Lithuania in 2017 [24], and more recently, a distinct MGF 505-9R/10R TRS variant was described in the Rome cluster in Italy during the 2023 epidemic wave, where it appears to be restricted to a locally circulating genotype II lineage [29]. Taken together, these observations suggest that the emergence and expansion of the MGF-5 lineage in Korean wild boars may have occurred independently within Korea; however, the possibility of international transmission cannot be completely excluded. Epidemiological differentiation of the ASFV is challenging when relying on a single genetic marker; therefore, multiple molecular markers are usually adopted to classify viral groups and analyze the distribution and spread patterns [24, 41]. However, some markers show little or no variation in specific regions, limiting their epidemiological resolution and indicating that marker combinations should be tailored to the regional context of each country [10, 54].

In Korea, ASFV strains identified in wild boars between 2019 and 2024 were classified into six clusters (Table 3) using the standard genotype marker p72, the subtype marker IGR, the Korea-specific marker MGF 360-1La, and the MGF 505-9R/10R marker originally reported in Europe. The spatiotemporal distribution of these six genetic clusters in Korean wild boars provides important clues for understanding how ASFV genotypes have been introduced and disseminated within the country. Meanwhile, recent spatial modeling studies have highlighted spatiotemporal heterogeneity in ASF risk and the effects of fencing in Korean wild boars [55, 56]. These findings suggest that the multi-marker genetic cluster analysis presented in this study could be used as a complementary tool to existing spatial risk assessments.

Clusters 1–3 appear to have originated along the northwestern Gyeonggi–Gangwon axis and subsequently spread into adjacent inland regions, whereas cluster 4 was first detected in the eastern coastal area of Gyeongbuk and then moved inland. Taken together, these non-overlapping geographic patterns suggest that the observed genetic diversity is more likely to reflect multiple introductions of already diversified genotype II ASFV lineages into different regions of Korea, followed by local circulation and spread, rather than gradual de novo evolution of a single domestic lineage over time. In contrast, cluster 2, although initially detected in the same Gyeonggi–northern Gangwon area as clusters 1–3, is characterized by a combination of molecular markers that has so far been reported only in Korean wild boars. This cluster was most frequently detected around 2021, when the number of ASFV-positive wild boars peaked nationwide. This temporal and spatial concentration pattern may indicate that, in this region, prolonged and intense circulation of ASFV allowed some degree of natural diversification to occur.

Overall, this study indicates that the ASFV epidemic among Korean wild boars began with the initial introduction of Genotype II viruses in 2019, followed by the subsequent emergence of multiple subtypes and clusters characterized by variations in the IGR and MGF regions. The spatiotemporal distribution of these clusters suggests that both the internal diversification of the initially introduced lineage over time within Korea and repeated introductions of genetically distinct viruses from external sources may have contributed to the observed genetic heterogeneity. Furthermore, to gain a more precise understanding of cluster-level transmission dynamics based on multiple molecular markers, future research should integrate annual and cluster-specific dissemination patterns with various environmental variables, such as seasonality, wild boar habitat density, and topographic or ecological factors. Because partial gene analyses may fail to detect rare or newly emerging variants, continuous mutation monitoring using whole-genome sequencing is required. Therefore, a surveillance strategy that combines multi-marker molecular analysis with whole-genome sequencing will play a crucial role in the early detection of emerging variants, elucidation of transmission dynamics, and strengthening of future control strategies.

## Supplementary Information

**Additional file 1.** **GenBank accession numbers of representative ASFV sequences.** This file contains the GenBank accession numbers of representative African swine fever virus sequences generated in this study.

## Data Availability

Representative African swine fever virus sequences generated in this study have been deposited in GenBank, and the accession numbers are provided in Additional file 1. The complete dataset of Sanger sequencing data is available from the corresponding author upon reasonable request.
