# Supplementary material for: Comprehensive molecular profiling of the African swine fever virus in Korean wild boars between 2019 and 2024
Source: Vet Res. 2026 May 13;57:73. doi: 10.1186/s13567-026-01723-z (PMC13173816; doi:10.1186/s13567-026-01723-z)
Supplement: Supplementary file 1 — Additional file 1. GenBank accession numbers of representative ASFV sequences. This file contains the GenBank accession numbers of representative African swine fever virus sequences generated in this study. [file 13567_2026_1723_MOESM1_ESM.docx]

**Additional file. List of GenBank accession numbers for representative ASFV sequences used in this study.**

| **Isolate name** | **Year** | **Region** | | **Marker** | **accession number** |
| --- | --- | --- | --- | --- | --- |
| South Korea/19S5464/Paju/2019/wildboar | 2019 | Paju | | p72 (B646L) | PX913416 |
| South Korea/19S804/Yeoncheon/2019/wildboar | 2019 | Yeoncheon | | p72 (B646L) | MN817977 |
| South Korea/19S3965/Paju/2019/wildboar | 2019 | Paju | | p72 (B646L) | PX913417 |
| South Korea/19S868/Cheorwon/2019/wildboar | 2019 | Cheorwon | | p72 (B646L) | PX913418 |
| South Korea/19S5311/Yeoncheon/2019/wildboar | 2019 | Yeoncheon | | p72 (B646L) | PX913419 |
| South Korea/19S5187/Cheorwon/2019/wildboar | 2019 | Cheorwon | | p72 (B646L) | PX913420 |
| South Korea/19S4918/Paju/2019/wildboar | 2019 | Paju | | p72 (B646L) | PX913421 |
| South Korea/20S8510/Inje/2020/wildboar | 2020 | Inje | | p72 (B646L) | PX913427 |
| South Korea/20S11902/Yeongwol/2020/wildboar | 2020 | Yeongwol | | p72 (B646L) | PX913422 |
| South Korea/20S137/Hwacheon/2020/wildboar | 2020 | Hwacheon | | p72 (B646L) | PX913423 |
| South Korea/20S29/Yeoncheon/2020/wildboar | 2020 | Yeoncheon | | p72 (B646L) | PX913424 |
| South Korea/20S2835/Paju/2020/wildboar | 2020 | Paju | | p72 (B646L) | PX913425 |
| South Korea/20S3315/Paju/2020/wildboar | 2020 | Paju | | p72 (B646L) | PX913426 |
| South Korea/20S10347/Chuncheon/2020/wildboar | 2020 | Chuncheon | | p72 (B646L) | PX914814 |
| South Korea/20S8519/Yanggu/2020/wildboar | 2020 | Yanggu | | p72 (B646L) | PX913428 |
| South Korea/20S8832/Pocheon/2020/wildboar | 2020 | Pocheon | | p72 (B646L) | PX913430 |
| South Korea/20S10787/Gapyeong/2020/wildboar | 2020 | Gapyeong | | p72 (B646L) | PX913429 |
| South Korea/21S4/Yeongwol/2021/wildboar | 2021 | Yeongwol | | p72 (B646L) | PX913432 |
| South Korea/21S185/Pocheon/2021/wildboar | 2021 | Pocheon | | p72 (B646L) | PX913431 |
| South Korea/21S1476/Gangneung/2021/wildboar | 2021 | Gangneung | | p72 (B646L) | PX913433 |
| South Korea/21S11135/Jeongseon/2021/wildboar | 2021 | Jeongseon | | p72 (B646L) | PX913434 |
| South Korea/21S15472/Danyang/2021/wildboar | 2021 | Danyang | | p72 (B646L) | PX913435 |
| South Korea/21S15031/Jecheon/2021/wildboar | 2021 | Jecheon | | p72 (B646L) | PX913436 |
| South Korea/21S15560/Chuncheon/2021/wildboar | 2021 | Chuncheon | | p72 (B646L) | PX913437 |
| South Korea/21S15940/Pyeongchang/2021/wildboar | 2021 | Pyeongchang | p72 (B646L) | | PX913438 |
| South Korea/21S16239/Yeongwol/2021/wildboar | 2021 | Yeongwol | | p72 (B646L) | PX913441 |
| South Korea/21S16333/Hongcheon/2021/wildboar | 2021 | Hongcheon | | p72 (B646L) | PX913442 |
| South Korea/22S1825/Sangju/2022/wildboar | 2022 | Sangju | | p72 (B646L) | PX913443 |
| South Korea/22S1305/Jecheon/2022/wildboar | 2022 | Jecheon | | p72 (B646L) | PX913444 |
| South Korea/22S2144/Hoengseong/2022/wildboar | 2022 | Hoengseong | | p72 (B646L) | PX913445 |
| South Korea/22S2607/Jeongseon/2022/wildboar | 2022 | Jeongseon | | p72 (B646L) | PX913446 |
| South Korea/22S2727/Boeun/2022/wildboar | 2022 | Boeun | | p72 (B646L) | PX913447 |
| South Korea/22S3161/Chungju/2022/wildboar | 2022 | Chungju | | p72 (B646L) | PX913448 |
| South Korea/22S14799/Samcheok/2022/wildboar | 2022 | Samcheok | | p72 (B646L) | PX913450 |
| South Korea/22S44283/Wonju/2022/wildboar | 2022 | Wonju | | p72 (B646L) | PX913449 |
| South Korea/22S43601/Mungyeong/2022/wildboar | 2022 | Mungyeong | | p72 (B646L) | PX913451 |
| South Korea/22S56962/Pyeongchang/2022/wildboar | 2022 | Pyeongchang | | p72 (B646L) | PX913452 |
| South Korea/23S6350/Yeongwol/2023/wildboar | 2023 | Yeongwol | | p72 (B646L) | PX913453 |
| South Korea/23S61376/Cheongsong/2023/wildboar | 2023 | Cheongsong | | p72 (B646L) | PX913454 |
| South Korea/23S63877/Pohang/2023/wildboar | 2023 | Pohang | | p72 (B646L) | PX913455 |
| South Korea/23S5689/Goesan/2023/wildboar | 2023 | Goesan | | p72 (B646L) | PX913456 |
| South Korea/23S9049/Sangju/2023/wildboar | 2023 | Sangju | | p72 (B646L) | PX913457 |
| South Korea/23S12918/Uljin/2023/wildboar | 2023 | Uljin | | p72 (B646L) | PX913458 |
| South Korea/23S35866/Yeongyang/2023/wildboar | 2023 | Yeongyang | | p72 (B646L) | PX913459 |
| South Korea/23S28484/Yeongdeok/2023/wildboar | 2023 | Yeongdeok | | p72 (B646L) | PX913460 |
| South Korea/23S68021/Cheongsong/2023/wildboar | 2023 | Cheongsong | | p72 (B646L) | PX913461 |
| South Korea/23S70088/Yeongju/2023/wildboar | 2023 | Yeongju | | p72 (B646L) | PX913462 |
| South Korea/24S1141/Uiseong/2024/wildboar | 2024 | Uiseong | | p72 (B646L) | PX913463 |
| South Korea/24S7635/Chungju/2024/wildboar | 2024 | Chungju | | p72 (B646L) | PX913464 |
| South Korea/24S4509/Busan/2024/wildboar | 2024 | Busan | | p72 (B646L) | PX913465 |
| South Korea/24S9910/Andong/2024/wildboar | 2024 | Andong | | p72 (B646L) | PX913466 |
| South Korea/24S12065/Danyang/2024/wildboar | 2024 | Danyang | | p72 (B646L) | PX914808 |
| South Korea/24S60419/Gunwi/2024/wildboar | 2024 | Gunwi | | p72 (B646L) | PX914809 |
| South Korea/24S69062/Jecheon/2024/wildboar | 2024 | Jecheon | | p72 (B646L) | PX914810 |
| South Korea/24S44796/Yanggu/2024/wildboar | 2024 | Yanggu | | p72 (B646L) | PX914811 |
| South Korea/24S19806/Pohang/2024/wildboar | 2024 | Pohang | | p72 (B646L) | PX914812 |
| South Korea/24S21140/Yecheon/2024/wildboar | 2024 | Yecheon | | p72 (B646L) | PX914813 |
| South Korea/19S5464/Paju/2019/wildboar | 2019 | Paju | | I73R/I329L | MT300325 |
| South Korea/19S804/Yeoncheon/2019/wildboar | 2019 | Yeoncheon | | I73R/I329L | MN817979 |
| South Korea/19S3965/Paju/2019/wildboar | 2019 | Paju | | I73R/I329L | MT300324 |
| South Korea/19S868/Cheorwon/2019/wildboar | 2019 | Cheorwon | | I73R/I329L | PX898606 |
| South Korea/19S5311/Yeoncheon/2019/wildboar | 2019 | Yeoncheon | | I73R/I329L | PX898607 |
| South Korea/19S5187/Cheorwon/2019/wildboar | 2019 | Cheorwon | | I73R/I329L | PX898608 |
| South Korea/19S4918/Paju/2019/wildboar | 2019 | Paju | | I73R/I329L | PX898609 |
| South Korea/20S8510/Inje/2020/wildboar | 2020 | Inje | | I73R/I329L | PX866895 |
| South Korea/20S11902/Yeongwol/2020/wildboar | 2020 | Yeongwol | | I73R/I329L | PX898610 |
| South Korea/20S137/Hwacheon/2020/wildboar | 2020 | Hwacheon | | I73R/I329L | PX898611 |
| South Korea/20S29/Yeoncheon/2020/wildboar | 2020 | Yeoncheon | | I73R/I329L | PX898612 |
| South Korea/20S2835/Paju/2020/wildboar | 2020 | Paju | | I73R/I329L | PX898644 |
| South Korea/20S3315/Paju/2020/wildboar | 2020 | Paju | | I73R/I329L | PX898645 |
| South Korea/20S10347/Chuncheon/2020/wildboar | 2020 | Chuncheon | | I73R/I329L | PX913467 |
| South Korea/20S8519/Yanggu/2020/wildboar | 2020 | Yanggu | | I73R/I329L | PX898614 |
| South Korea/20S8832/Pocheon/2020/wildboar | 2020 | Pocheon | | I73R/I329L | PX898615 |
| South Korea/20S10787/Gapyeong/2020/wildboar | 2020 | Gapyeong | | I73R/I329L | PX898616 |
| South Korea/21S4/Yeongwol/2021/wildboar | 2021 | Yeongwol | | I73R/I329L | PX898617 |
| South Korea/21S185/Pocheon/2021/wildboar | 2021 | Pocheon | | I73R/I329L | PX898618 |
| South Korea/21S1476/Gangneung/2021/wildboar | 2021 | Gangneung | | I73R/I329L | PX898619 |
| South Korea/21S11135/Jeongseon/2021/wildboar | 2021 | Jeongseon | | I73R/I329L | PX898620 |
| South Korea/21S15472/Danyang/2021/wildboar | 2021 | Danyang | | I73R/I329L | PX898621 |
| South Korea/21S15031/Jecheon/2021/wildboar | 2021 | Jecheon | | I73R/I329L | PX898622 |
| South Korea/21S15560/Chuncheon/2021/wildboar | 2021 | Chuncheon | | I73R/I329L | PX898623 |
| South Korea/21S15940/Pyeongchang/2021/wildboar | 2021 | Pyeongchang | | I73R/I329L | PX898624 |
| South Korea/21S16239/Yeongwol/2021/wildboar | 2021 | Yeongwol | | I73R/I329L | PX898625 |
| South Korea/21S16333/Hongcheon/2021/wildboar | 2021 | Hongcheon | | I73R/I329L | PX898626 |
| South Korea/22S1825/Sangju/2022/wildboar | 2022 | Sangju | | I73R/I329L | PX898627 |
| South Korea/22S1305/Jecheon/2022/wildboar | 2022 | Jecheon | | I73R/I329L | PX898628 |
| South Korea/22S2144/Hoengseong/2022/wildboar | 2022 | Hoengseong | | I73R/I329L | PX898629 |
| South Korea/22S2607/Jeongseon/2022/wildboar | 2022 | Jeongseon | | I73R/I329L | PX898630 |
| South Korea/22S2727/Boeun/2022/wildboar | 2022 | Boeun | | I73R/I329L | PX898631 |
| South Korea/22S3161/Chungju/2022/wildboar | 2022 | Chungju | | I73R/I329L | PX898632 |
| South Korea/22S14799/Samcheok/2022/wildboar | 2022 | Samcheok | | I73R/I329L | PX898633 |
| South Korea/22S44283/Wonju/2022/wildboar | 2022 | Wonju | | I73R/I329L | PX898634 |
| South Korea/22S43601/Mungyeong/2022/wildboar | 2022 | Mungyeong | | I73R/I329L | PX898635 |
| South Korea/22S56962/Pyeongchang/2022/wildboar | 2022 | Pyeongchang | | I73R/I329L | PX898636 |
| South Korea/23S6350/Yeongwol/2023/wildboar | 2023 | Yeongwol | | I73R/I329L | PX898637 |
| South Korea/23S61376/Cheongsong/2023/wildboar | 2023 | Cheongsong | | I73R/I329L | PX898646 |
| South Korea/23S63877/Pohang/2023/wildboar | 2023 | Pohang | | I73R/I329L | PX898647 |
| South Korea/23S5689/Goesan/2023/wildboar | 2023 | Goesan | | I73R/I329L | PX898638 |
| South Korea/23S9049/Sangju/2023/wildboar | 2023 | Sangju | | I73R/I329L | PX898639 |
| South Korea/23S12918/Uljin/2023/wildboar | 2023 | Uljin | | I73R/I329L | PX898640 |
| South Korea/23S35866/Yeongyang/2023/wildboar | 2023 | Yeongyang | | I73R/I329L | PX898641 |
| South Korea/23S28484/Yeongdeok/2023/wildboar | 2023 | Yeongdeok | | I73R/I329L | PX898642 |
| South Korea/23S68021/Cheongsong/2023/wildboar | 2023 | Cheongsong | | I73R/I329L | PX898648 |
| South Korea/23S70088/Yeongju/2023/wildboar | 2023 | Yeongju | | I73R/I329L | PX898649 |
| South Korea/24S1141/Uiseong/2024/wildboar | 2024 | Uiseong | | I73R/I329L | PX898643 |
| South Korea/24S7635/Chungju/2024/wildboar | 2024 | Chungju | | I73R/I329L | PX898650 |
| South Korea/24S4509/Busan/2024/wildboar | 2024 | Busan | | I73R/I329L | PX898651 |
| South Korea/24S9910/Andong/2024/wildboar | 2024 | Andong | | I73R/I329L | PX898652 |
| South Korea/24S12065/Danyang/2024/wildboar | 2024 | Danyang | | I73R/I329L | PX898653 |
| South Korea/24S60419/Gunwi/2024/wildboar | 2024 | Gunwi | | I73R/I329L | PX898654 |
| South Korea/24S69062/Jecheon/2024/wildboar | 2024 | Jecheon | | I73R/I329L | PX898655 |
| South Korea/24S44796/Yanggu/2024/wildboar | 2024 | Yanggu | | I73R/I329L | PX898656 |
| South Korea/24S19806/Pohang/2024/wildboar | 2024 | Pohang | | I73R/I329L | PX898657 |
| South Korea/24S21140/Yecheon/2024/wildboar | 2024 | Yecheon | | I73R/I329L | PX898658 |
| South Korea/19S5464/Paju/2019/wildboar | 2019 | Paju | | MGF 360-1La | PX930647 |
| South Korea/19S804/Yeoncheon/2019/wildboar | 2019 | Yeoncheon | | MGF 360-1La | PX930648 |
| South Korea/19S3965/Paju/2019/wildboar | 2019 | Paju | | MGF 360-1La | PX930649 |
| South Korea/19S868/Cheorwon/2019/wildboar | 2019 | Cheorwon | | MGF 360-1La | PX930650 |
| South Korea/19S5311/Yeoncheon/2019/wildboar | 2019 | Yeoncheon | | MGF 360-1La | PX930651 |
| South Korea/19S5187/Cheorwon/2019/wildboar | 2019 | Cheorwon | | MGF 360-1La | PX930652 |
| South Korea/19S4918/Paju/2019/wildboar | 2019 | Paju | | MGF 360-1La | PX930653 |
| South Korea/20S8510/Inje/2020/wildboar | 2020 | Inje | | MGF 360-1La | PX930694 |
| South Korea/20S11902/Yeongwol/2020/wildboar | 2020 | Yeongwol | | MGF 360-1La | PX930654 |
| South Korea/20S137/Hwacheon/2020/wildboar | 2020 | Hwacheon | | MGF 360-1La | PX930655 |
| South Korea/20S29/Yeoncheon/2020/wildboar | 2020 | Yeoncheon | | MGF 360-1La | PX930656 |
| South Korea/20S2835/Paju/2020/wildboar | 2020 | Paju | | MGF 360-1La | PX930657 |
| South Korea/20S3315/Paju/2020/wildboar | 2020 | Paju | | MGF 360-1La | PX930658 |
| South Korea/20S10347/Chuncheon/2020/wildboar | 2020 | Chuncheon | | MGF 360-1La | PX930659 |
| South Korea/20S8519/Yanggu/2020/wildboar | 2020 | Yanggu | | MGF 360-1La | PX930695 |
| South Korea/20S8832/Pocheon/2020/wildboar | 2020 | Pocheon | | MGF 360-1La | PX930660 |
| South Korea/20S10787/Gapyeong/2020/wildboar | 2020 | Gapyeong | | MGF 360-1La | PX930661 |
| South Korea/21S4/Yeongwol/2021/wildboar | 2021 | Yeongwol | | MGF 360-1La | PX930696 |
| South Korea/21S185/Pocheon/2021/wildboar | 2021 | Pocheon | | MGF 360-1La | PX930663 |
| South Korea/21S1476/Gangneung/2021/wildboar | 2021 | Gangneung | | MGF 360-1La | PX930697 |
| South Korea/21S11135/Jeongseon/2021/wildboar | 2021 | Jeongseon | | MGF 360-1La | PX930662 |
| South Korea/21S15472/Danyang/2021/wildboar | 2021 | Danyang | | MGF 360-1La | PX930703 |
| South Korea/21S15031/Jecheon/2021/wildboar | 2021 | Jecheon | | MGF 360-1La | PX930664 |
| South Korea/21S15560/Chuncheon/2021/wildboar | 2021 | Chuncheon | | MGF 360-1La | PX930665 |
| South Korea/21S15940/Pyeongchang/2021/wildboar | 2021 | Pyeongchang | | MGF 360-1La | PX930666 |
| South Korea/21S16239/Yeongwol/2021/wildboar | 2021 | Yeongwol | | MGF 360-1La | PX930667 |
| South Korea/21S16333/Hongcheon/2021/wildboar | 2021 | Hongcheon | | MGF 360-1La | PX930668 |
| South Korea/22S1825/Sangju/2022/wildboar | 2022 | Sangju | | MGF 360-1La | PX930669 |
| South Korea/22S1305/Jecheon/2022/wildboar | 2022 | Jecheon | | MGF 360-1La | PX930698 |
| South Korea/22S2144/Hoengseong/2022/wildboar | 2022 | Hoengseong | | MGF 360-1La | PX930670 |
| South Korea/22S2607/Jeongseon/2022/wildboar | 2022 | Jeongseon | | MGF 360-1La | PX930671 |
| South Korea/22S2727/Boeun/2022/wildboar | 2022 | Boeun | | MGF 360-1La | PX930672 |
| South Korea/22S3161/Chungju/2022/wildboar | 2022 | Chungju | | MGF 360-1La | PX930673 |
| South Korea/22S14799/Samcheok/2022/wildboar | 2022 | Samcheok | | MGF 360-1La | PX930699 |
| South Korea/22S44283/Wonju/2022/wildboar | 2022 | Wonju | | MGF 360-1La | PX930674 |
| South Korea/22S43601/Mungyeong/2022/wildboar | 2022 | Mungyeong | | MGF 360-1La | PX930675 |
| South Korea/22S56962/Pyeongchang/2022/wildboar | 2022 | Pyeongchang | | MGF 360-1La | PX930676 |
| South Korea/23S6350/Yeongwol/2023/wildboar | 2023 | Yeongwol | | MGF 360-1La | PX930677 |
| South Korea/23S61376/Cheongsong/2023/wildboar | 2023 | Cheongsong | | MGF 360-1La | PX930678 |
| South Korea/23S63877/Pohang/2023/wildboar | 2023 | Pohang | | MGF 360-1La | PX930679 |
| South Korea/23S5689/Goesan/2023/wildboar | 2023 | Goesan | | MGF 360-1La | PX930680 |
| South Korea/23S9049/Sangju/2023/wildboar | 2023 | Sangju | | MGF 360-1La | PX930681 |
| South Korea/23S12918/Uljin/2023/wildboar | 2023 | Uljin | | MGF 360-1La | PX930682 |
| South Korea/23S35866/Yeongyang/2023/wildboar | 2023 | Yeongyang | | MGF 360-1La | PX930683 |
| South Korea/23S28484/Yeongdeok/2023/wildboar | 2023 | Yeongdeok | | MGF 360-1La | PX930684 |
| South Korea/23S68021/Cheongsong/2023/wildboar | 2023 | Cheongsong | | MGF 360-1La | PX930685 |
| South Korea/23S70088/Yeongju/2023/wildboar | 2023 | Yeongju | | MGF 360-1La | PX930686 |
| South Korea/24S1141/Uiseong/2024/wildboar | 2024 | Uiseong | | MGF 360-1La | PX930687 |
| South Korea/24S7635/Chungju/2024/wildboar | 2024 | Chungju | | MGF 360-1La | PX930688 |
| South Korea/24S4509/Busan/2024/wildboar | 2024 | Busan | | MGF 360-1La | PX930689 |
| South Korea/24S9910/Andong/2024/wildboar | 2024 | Andong | | MGF 360-1La | PX930690 |
| South Korea/24S12065/Danyang/2024/wildboar | 2024 | Danyang | | MGF 360-1La | PX930700 |
| South Korea/24S60419/Gunwi/2024/wildboar | 2024 | Gunwi | | MGF 360-1La | PX930691 |
| South Korea/24S69062/Jecheon/2024/wildboar | 2024 | Jecheon | | MGF 360-1La | PX930701 |
| South Korea/24S44796/Yanggu/2024/wildboar | 2024 | Yanggu | | MGF 360-1La | PX930702 |
| South Korea/24S19806/Pohang/2024/wildboar | 2024 | Pohang | | MGF 360-1La | PX930692 |
| South Korea/24S21140/Yecheon/2024/wildboar | 2024 | Yecheon | | MGF 360-1La | PX930693 |
| South Korea/19S5464/Paju/2019/wildboar | 2019 | Paju | | MGF 505-9R/10R | PX967172 |
| South Korea/19S804/Yeoncheon/2019/wildboar | 2019 | Yeoncheon | | MGF 505-9R/10R | PX967173 |
| South Korea/19S3965/Paju/2019/wildboar | 2019 | Paju | | MGF 505-9R/10R | PX967174 |
| South Korea/19S868/Cheorwon/2019/wildboar | 2019 | Cheorwon | | MGF 505-9R/10R | PX967209 |
| South Korea/19S5311/Yeoncheon/2019/wildboar | 2019 | Yeoncheon | | MGF 505-9R/10R | PX967175 |
| South Korea/19S5187/Cheorwon/2019/wildboar | 2019 | Cheorwon | | MGF 505-9R/10R | PX967176 |
| South Korea/19S4918/Paju/2019/wildboar | 2019 | Paju | | MGF 505-9R/10R | PX967177 |
| South Korea/20S8510/Inje/2020/wildboar | 2020 | Inje | | MGF 505-9R/10R | PX967178 |
| South Korea/20S11902/Yeongwol/2020/wildboar | 2020 | Yeongwol | | MGF 505-9R/10R | PX967179 |
| South Korea/20S137/Hwacheon/2020/wildboar | 2020 | Hwacheon | | MGF 505-9R/10R | PX967180 |
| South Korea/20S29/Yeoncheon/2020/wildboar | 2020 | Yeoncheon | | MGF 505-9R/10R | PX967181 |
| South Korea/20S2835/Paju/2020/wildboar | 2020 | Paju | | MGF 505-9R/10R | PX967182 |
| South Korea/20S3315/Paju/2020/wildboar | 2020 | Paju | | MGF 505-9R/10R | PX967183 |
| South Korea/20S10347/Chuncheon/2020/wildboar | 2020 | Chuncheon | | MGF 505-9R/10R | PX967184 |
| South Korea/20S8519/Yanggu/2020/wildboar | 2020 | Yanggu | | MGF 505-9R/10R | PX967185 |
| South Korea/20S8832/Pocheon/2020/wildboar | 2020 | Pocheon | | MGF 505-9R/10R | PX967186 |
| South Korea/20S10787/Gapyeong/2020/wildboar | 2020 | Gapyeong | | MGF 505-9R/10R | PX967187 |
| South Korea/21S4/Yeongwol/2021/wildboar | 2021 | Yeongwol | | MGF 505-9R/10R | PX967210 |
| South Korea/21S185/Pocheon/2021/wildboar | 2021 | Pocheon | | MGF 505-9R/10R | PX967188 |
| South Korea/21S1476/Gangneung/2021/wildboar | 2021 | Gangneung | | MGF 505-9R/10R | PX967189 |
| South Korea/21S11135/Jeongseon/2021/wildboar | 2021 | Jeongseon | | MGF 505-9R/10R | PX967190 |
| South Korea/21S15472/Danyang/2021/wildboar | 2021 | Danyang | | MGF 505-9R/10R | PX967191 |
| South Korea/21S15031/Jecheon/2021/wildboar | 2021 | Jecheon | | MGF 505-9R/10R | PX967211 |
| South Korea/21S15560/Chuncheon/2021/wildboar | 2021 | Chuncheon | | MGF 505-9R/10R | PX967212 |
| South Korea/21S15940/Pyeongchang/2021/wildboar | 2021 | Pyeongchang | | MGF 505-9R/10R | PX967212 |
| South Korea/21S16239/Yeongwol/2021/wildboar | 2021 | Yeongwol | | MGF 505-9R/10R | PX967225 |
| South Korea/21S16333/Hongcheon/2021/wildboar | 2021 | Hongcheon | | MGF 505-9R/10R | PX967214 |
| South Korea/22S1825/Sangju/2022/wildboar | 2022 | Sangju | | MGF 505-9R/10R | PX967226 |
| South Korea/22S1305/Jecheon/2022/wildboar | 2022 | Jecheon | | MGF 505-9R/10R | PX967215 |
| South Korea/22S2144/Hoengseong/2022/wildboar | 2022 | Hoengseong | | MGF 505-9R/10R | PX967227 |
| South Korea/22S2607/Jeongseon/2022/wildboar | 2022 | Jeongseon | | MGF 505-9R/10R | PX967228 |
| South Korea/22S2727/Boeun/2022/wildboar | 2022 | Boeun | | MGF 505-9R/10R | PX967229 |
| South Korea/22S3161/Chungju/2022/wildboar | 2022 | Chungju | | MGF 505-9R/10R | PX967216 |
| South Korea/22S14799/Samcheok/2022/wildboar | 2022 | Samcheok | | MGF 505-9R/10R | PX967217 |
| South Korea/22S44283/Wonju/2022/wildboar | 2022 | Wonju | | MGF 505-9R/10R | PX967218 |
| South Korea/22S43601/Mungyeong/2022/wildboar | 2022 | Mungyeong | | MGF 505-9R/10R | PX967230 |
| South Korea/22S56962/Pyeongchang/2022/wildboar | 2022 | Pyeongchang | | MGF 505-9R/10R | PX967231 |
| South Korea/23S6350/Yeongwol/2023/wildboar | 2023 | Yeongwol | | MGF 505-9R/10R | PX967232 |
| South Korea/23S61376/Cheongsong/2023/wildboar | 2023 | Cheongsong | | MGF 505-9R/10R | PX967233 |
| South Korea/23S63877/Pohang/2023/wildboar | 2023 | Pohang | | MGF 505-9R/10R | PX967234 |
| South Korea/23S5689/Goesan/2023/wildboar | 2023 | Goesan | | MGF 505-9R/10R | PX967219 |
| South Korea/23S9049/Sangju/2023/wildboar | 2023 | Sangju | | MGF 505-9R/10R | PX967220 |
| South Korea/23S12918/Uljin/2023/wildboar | 2023 | Uljin | | MGF 505-9R/10R | PX967235 |
| South Korea/23S35866/Yeongyang/2023/wildboar | 2023 | Yeongyang | | MGF 505-9R/10R | PX967236 |
| South Korea/23S28484/Yeongdeok/2023/wildboar | 2023 | Yeongdeok | | MGF 505-9R/10R | PX967237 |
| South Korea/23S68021/Cheongsong/2023/wildboar | 2023 | Cheongsong | | MGF 505-9R/10R | PX967238 |
| South Korea/23S70088/Yeongju/2023/wildboar | 2023 | Yeongju | | MGF 505-9R/10R | PX967239 |
| South Korea/24S1141/Uiseong/2024/wildboar | 2024 | Uiseong | | MGF 505-9R/10R | PX967240 |
| South Korea/24S7635/Chungju/2024/wildboar | 2024 | Chungju | | MGF 505-9R/10R | PX967221 |
| South Korea/24S4509/Busan/2024/wildboar | 2024 | Busan | | MGF 505-9R/10R | PX967241 |
| South Korea/24S9910/Andong/2024/wildboar | 2024 | Andong | | MGF 505-9R/10R | PX967242 |
| South Korea/24S12065/Danyang/2024/wildboar | 2024 | Danyang | | MGF 505-9R/10R | PX967222 |
| South Korea/24S60419/Gunwi/2024/wildboar | 2024 | Gunwi | | MGF 505-9R/10R | PX967243 |
| South Korea/24S69062/Jecheon/2024/wildboar | 2024 | Jecheon | | MGF 505-9R/10R | PX967223 |
| South Korea/24S44796/Yanggu/2024/wildboar | 2024 | Yanggu | | MGF 505-9R/10R | PX967224 |
| South Korea/24S19806/Pohang/2024/wildboar | 2024 | Pohang | | MGF 505-9R/10R | PX967244 |
| South Korea/24S21140/Yecheon/2024/wildboar | 2024 | Yecheon | | MGF 505-9R/10R | PX967245 |
